# Is There a Correlation between Vitamin C Status and Catecholamines Concentrations in Hemodialysis Patients?

**Published:** 2008-06

**Authors:** Isaline Coquet, Jean-Marc Doise, Jean-Claude Guilland, Catherine Vergely, Christiane Mousson, Luc Rochette

**Affiliations:** 1 *Service de Réanimation Médicale, Centre Hospitalier Universitaire, 2 boulevard Maréchal de Lattre de Tassigny, BP 77908, 21079 Dijon Cedex, France;*; 2 *L.P.P.C.E., Facultés de Médecine et Pharmacie, 7 boulevard Jeanne d’Arc, 21033 Dijon Cedex, France;*; 3 *Service de Néphrologie, Centre Hospitalier Universitaire, 2 boulevard Maréchal de Lattre de Tassigny, 21079 Dijon Cedex, France*

**Keywords:** ascorbyl free radical, catecholamines, haemodialysis, oxidative stress, vitamin C

## Abstract

It is well established that there is a high incidence of cardiovascular diseases in hemodialysis patients, and involvement of oxidative stress has been hypothesised in these phenomena. Plasma norepinephrine is an independent predictor of many causes of mortality in general, and high norepinephrine levels predict cardiovascular complications in end stage renal disease. The aim of our study was to evaluate the potential link between vitamin C status, a marker of oxidative stress, and catecholamine concentrations before and after hemodialysis sessions. In a prospective study of 16 chronic hemodialysis patients, ascorbyl free radical levels were directly measured using electron spin resonance spectroscopy. These values were expressed with respect to vitamin C concentrations to obtain a direct index of oxidative stress. Vitamin C, epinephrine and norepinephrine were measured by high performance liquid chromatography. The data were examined for correlations between these compounds and clinical parameters including blood pressure and heart rates. In hemodialysis patients, ascorbyl free radical/vitamin C ratios increased significantly after dialysis. No differences were observed for catecholamine concentrations during hemodialysis sessions. In multivariate analysis, the ascorbyl free radical/vitamin C ratio did not correlate with epinephrine or norepinephrine levels. In our study, plasma norepinephrine and ascorbyl free radical/vitamin C ratios were not related among patients with end-stage renal disease. From these findings, we conclude that although these two factors are likely to be involved in the same causal pathway leading to cardiovascular events, it is likely that they seem to be independent.

## INTRODUCTION

Patients on chronic hemodialysis (HD) treatment have a fourfold mortality compared with general population, mainly attributed to cardiovascular diseases (1). Oxidative stress seems to contribute to the high prevalence of cardiovascular disease in these patients (2). Moreover, major antioxidants as vitamin C are decreased by chronic renal failure (CRF) and HD sessions (3). These synergistic situations exacerbate vitamin C deficiency. Among emerging risk factors in end-stage renal disease (ESRD), epinephrine (E) levels seem to be of major interest. Norepinephrine (NE) concentrations predict adverse cardiovascular outcomes among HD patients and are strongly associated with left ventricular concentric hypertrophy and left ventricular dysfunction and could underlie common mechanisms leading to cardiovascular complications (4-6). Therefore, an analysis of the relationship between plasma catecholamines and vitamin C status, a marker of oxidative stress, may provide useful clues for understanding of mechanisms mediating the adverse effects of risk factors in ESRD. The present study evaluates direct index of oxidative stress: ascorbyl free radical (AFR) detected by electron spin resonance spectroscopy (ESR) correlate with catecholamines concentrations during HD session.

## MATERIAL AND METHODS

### Patients and methods

The protocol was in conformity with ethical guidelines of our institutions, and informed consent was obtained from each participant. Sixteen patients (female: 10, male: 6) with CRF who had undergone maintenance HD for >3 months were studied. All patients underwent dialysis three times weekly for 4 hours to maintain a minimum Kt/V urea index of 1.2 per session. None had any concurrent illnesses at the time of the study nor were they taking supplemental ascorbic acid, iron or rhu-EPO. Seven HD patients had antihypertensive therapies: calcium inhibitor (n=6) or diuretics (n=1). Treatments with beta-blockers and ACE inhibitors were terminated one month prior to the study and replaced by a calcium-inhibitor (Amlodipine, Pfizer Lab. Paris). Patients were dialysed with biocompatible Polyacrylonitrile membrane (AN69, Hospal^®^). All patients received bicarbonate dialysis, and sessions were conducted under standard conditions in the same unit, with sterile apyrogen delivered water. Blood and dialysate flow-rates were maintained at 250 ml/min and 500 ml/min respectively. The final dialysate concentration was: HCO_3_^-^ 35 mM, Na^+^ 140 mM, K^+^ 1.5-2 mM, Ca^2+^ 1.5 mM. Patients received continuous infusion of heparin (n=10) or bolus of low molecular weight heparin (n=6).

### Clinical measurements

Blood pressure was monitored via a sphygmomanometer (Nippo Collin electronics-Press Mate-BP 8800C^®^, Japan) and checked every hour during each HD session. Cardiac frequency was continuously monitored during HD session. All patients were weighed before and after HD session.

### Biochemical measurements

Arterial blood samples were collected before and after HD session. Samples were immediately centrifuged. Fifty microliters of plasma were mixed with 100 microliters of 5% metaphosphoric acid. This solution was aliquoted and stored at -80°C until assayed.

### Electron spin resonance spectroscopy measurements

ESR spectra were recorded on a Bruker ESP 300E-X^®^ band spectrometer (Wissembourg, France). Plasma samples for AFR measurements were introduced into an aqueous ESR quartz flat cell, and ESR spectra were recorded at room temperature using a TM110 cavity. Spectrometer settings for the detection of AFR have already been described (7). Relative radical concentrations (arbitrary units: AU) were determined by measurement of line intensities on spectra recorded with identical spectrometer settings (8). AFR was expressed as AU, and as AU relative to plasmatic vitamin C concentration.

### Metabolic measurements

Plasma vitamin C concentration (mg/l) and catecholamines (E, NE) were measured by high performance liquid chromatography (HPLC) (9, 10).

### Statistical analysis

Data were compared using a one-way analysis of variance. Differences between values before and after the HD session were evaluated by the Wilcoxon test. Correlations between variables were evaluated by the Spearman test. For semi-quantitative variables, Kruskal-Wallis test was used. A *p* value <0.05 was considered statistically significant. Data were presented as mean ± SEM.

## RESULTS AND DISCUSSION

The main characteristics of the 16 HD patients are presented in Table 1. No adverse clinical events occurred during the HD sessions. Systolic blood pressure declined significantly during HD session (135 vs 120 mmHg). No significant difference in terms of diastolic blood pressure measures, or heart rates were found between Pre-HD and Post-HD session. Proteins were significantly increased after dialysis sessions (69.38 ± 3.2 g/l pre-HD vs. 80.19 ± 2.6 g/l post-HD, *p*<0.05). Catecholamines concentrations were comparable during Pre-HD and Post-HD session.

**Table 1:** Main characteristics of hemodialysis patients

Mean age ± SD (years)	66 ±16.2
Sex (men/women)	6/10
Renal diseases	
Glomerulonephritis	4
Interstitial nephropathy	1
Nephroangiosclerosis	3
Diabetes mellitus	2
Polycystic kidney disease	1
Myeloma	1
Neoplasm	1
Cystinuria-Lysinuric	1
Berger disease	1
Goodpasture syndrome	1
Duration of follow-up dialysis (years)	6.28 ± 1.41
Kt/V	1.39 ± 0.09

Table 2 presents vitamin C status in HD patients before (pre-HD) and after (post-HD) HD sessions. The HD procedure induced a decrease of plasma vitamin C concentration (53.18 ± 55.24 mg/l pre-HD vs. 24.65 ± 16.78 mg/l, *p*<0.05), associated with an increase AFR/ vitamin C ratio (0.58 ± 0.48 pre-HD vs. 0.67 ± 0.49 au/mg/l post-HD, *p*<0.05). Hemoconcentration adjustment relative to post-HD plasma protein level did not change the interpretation of results. No correlations between AFR/vitamin C ratio and NE or E concentrations, before or after HD session were demonstrated.

**Table 2:** Variations of vitamin C status, ascorbyl free radicals and catecholamines (Noradrenaline and Adrenaline) concentrations during hemodialysis (HD) session

	Before HD session	After HD session	*p*

Plasma Vitamin C (mg/l)	53.18 ± 55.24	24.65 ± 16.78	<0.001
Ascorbyl free radical (AU)	17.38 ± 11.10	11.75 ± 2.72	<0.05
AFR/vitamin C (AU)	0.58 ± 0.48	0.67 ± 0.49	<0.05
Noradrenaline (ng/ml)	0.41 ± 0.16	0.51 ± 0.23	NS
Adrenaline (ng/ml)	0.67 ± 0.38	0.67 ± 0.48	NS

AFR, Ascorbyl Free Radical; NS, statistically not significant differences. *p*>0.05.

This study reveals that AFR/vitamin C ratio is not correlated with E or NE levels; it is likely that they seem to be independent. Plasma NE levels were increased for a substantial proportion of patients with ESRD. There is indirect evidence that increased activity of the sympathetic nervous system might contribute to hypertension in patients with ESRD. Converse *et al*. (11) showed that the increased sympathetic nervous discharge in HD patients was constant throughout the interdialytic intervals, despite large variations in volume and blood pressure. It is still a matter of debate whether the changes in plasma catecholamines result from decreased metabolic clearance, decreased neuronal reuptake or increased sympathetic nerve discharge (12). In contrast, previous investigations on catecholamines have shown conflicting results but these studies did not include the gold standard of cardiac autonomic nervous system activity which could influence catecholamine levels (4, 5). Catecholamines are small dialyzable molecules and their removal during HD could be responsible for the “non-response” observed during standard HD. Zocalli *et al*. (4) showed that plasma NE exceeded the upper limit of normal range in 102 dialysis patients and was independent predictor of mortality but high NE levels predict cardiovascular events in ESRD patients.

The present study confirms that chronic renal failure is associated with a transient increase in the oxidant state of plasma, demonstrated by the presence of products associated with free radical activity. AFR detected by ESR is derived directly from the oxidation of vitamin C. Therefore AFR/ vitamin C ratio can be considered as a reliable non-invasive marker of free radical oxidation. The increase of AFR/vi C ratio observed in our study seems to reflect an increase in oxidation of vitamin C associated to the HD. Our results are in accordance with previous studies (13-15). Despite the use of polyacrylonitrile high biocompatible membranes, HD induces inflammation and neutrophil activation, and is associated with free radical production (16). Decreased plasma vitamin C concentrations observed after dialysis sessions could be contributed to by filtration of low-molecular-weight compound through this membrane.

HD is correlated with a higher AFR/ vitamin C ratio; this suggests that increased lengths of HD might stimulate oxidative stress. This hypothesis is consistent with observations from other studies suggesting that uraemia and long-term HD are associated with oxidative stress (17). In addition to spontaneous and irreversible loss of ascorbate, erythrocytes can take up dehydroascorbate using the glucose transporter 1 (GLUT 1) and can reduce this to ascorbate. Recently, Wann *et al* (18) reported that intraerythrocyte ascorbate concentrations decreased after one HD session compared with Pre-HD, and that they recovered to Pre-HD levels two days later. Enhanced GLUT 1 expression on erythrocytes membranes for HD patients may contribute to better preservation of intracellular ascorbate compared with healthy subjects. In our study, we observed no association between plasma NE and AFR/vitamin C ratio. More recently, Mallamaci *et al*. (19) analysed the relationships between NE and asymmetric dimethylarginine (ADMA) levels, a marker of oxidative stress, and an important inhibitor of NO synthesis in ESRD patients. Plasma ADMA levels were strongly associated with plasma NE levels. However, multivariate analysis indicated NE to be an independent predictor of death or cardiac events, while plasma ADMA levels emerged as a highly significant predictor of those outcomes.

The role of NO in HD is still debated. Some reports demonstrated a correlation between NO level, Pre-HD blood pressure of patients and dialysis hypotensive episodes during the HD session (20). Nishimura *et al*. (21) investigated the possible involvement of NO in acute hypotension during maintenance HD and measured the plasma concentrations of nitrate anion, a stable metabolite of NO. Their results suggested that inhibited sympathetic activity was one of the causes of acute hypotension during dialysis and that production of NO was implicated in this inhibition of the sympathetic activity. On the other hand, NO reacts with superoxide radical yielding to peroynitrite, which is a potent oxidant; thus, this compound has been proposed to play a role in the pathogenesis of diseases implicating endothelial cells (21-23). In a recent study, examining ESRD patients on hemodialysis, it was reported that the antioxidant calcium channel blocker amlodipine significantly reduced oxidative stress indices contributing to cardiovascular disease (24).

In conclusion, our study confirms the presence of oxidative stress during HD, despite the use of high compatible membranes. No correlation between oxidative stress and variations in plasma catecholamines levels was demonstrated. Repetitive free radical aggression during maintenance HD could induce membrane peroxidation and initiate endothelial injury, and thus may contribute to cardiovascular complications in this population.
